# C-peptide, Na^+^,K^+^-ATPase, and Diabetes

**DOI:** 10.1080/15438600490424514

**Published:** 2004

**Authors:** P. Vague, T. C. Coste, M. F. Jannot, D. Raccah, M. Tsimaratos

**Affiliations:** 1 Département de Nutrition-Endocrinologie-Maladies Métaboliques CHU Timone Boulevard Jean Moulin Marseille Cedex 05 13385 France

## Abstract

Na^+^,K^+^-ATPase is an ubiquitous membrane enzyme that allows the extrusion of three sodium ions from the cell and two potassium ions from the extracellular fluid. Its activity is decreased in many tissues of streptozotocin-induced diabetic animals. This impairment could be at least partly responsible for the development of diabetic complications. Na^+^,K^+^-ATPase activity is decreased in the red blood cell membranes of type 1 diabetic individuals, irrespective of the degree of diabetic control. It is less impaired or even normal in those of type 2 diabetic patients. The authors have shown that in the red blood cells of type 2 diabetic patients, Na^+^,K^+^-ATPase activity was strongly related to blood C-peptide levels in non–insulin-treated patients (in whom C-peptide concentration reflects that of insulin) as well as in insulin-treated patients. Furthermore, a gene-environment relationship has been observed. The alpha-1 isoform of the enzyme predominant in red blood cells and nerve tissue is encoded by the *ATP1A1* gene.Apolymorphism in the intron 1 of this gene is associated with lower enzyme activity in patients with C-peptide deficiency either with type 1 or type 2 diabetes, but not in normal individuals. There are several lines of evidence for a low C-peptide level being responsible for low Na^+^,K^+^-ATPase activity in the red blood cells. Short-term C-peptide infusion to type 1 diabetic patients restores normal Na^+^,K^+^-ATPase activity. Islet transplantation, which restores endogenous C-peptide secretion, enhances Na^+^,K^+^-ATPase activity proportionally to the rise in C-peptide. This C-peptide effect is not indirect. In fact, incubation of diabetic red blood cells with C-peptide at physiological concentration leads to an increase of Na^+^,K^+^-ATPase activity. In isolated proximal tubules of rats or in the medullary thick ascending limb of the kidney, C-peptide stimulates in a dose-dependent manner Na^+^,K^+^-ATPase activity. This impairment in Na^+^,K^+^-ATPase activity, mainly secondary to the lack of C-peptide, plays probably a role in the development of diabetic complications. Arguments have been developed showing that the diabetesinduced decrease in Na^+^,K^+^-ATPase activity compromises microvascular blood flow by two mechanisms: by affecting microvascular regulation and by decreasing red blood cell deformability, which leads to an increase in blood viscosity. C-peptide infusion restores red blood cell deformability and microvascular blood flow concomitantly with Na^+^,K^+^-ATPase activity. The defect in ATPase is strongly related to diabetic neuropathy. Patients with neuropathy have lower ATPase activity than those without. The diabetes-induced impairment in Na^+^,K^+^-ATPase activity is identical in red blood cells and neural tissue. Red blood cell ATPase activity is related to nerve conduction velocity in the peroneal and the tibial nerve of diabetic patients. C-peptide infusion to diabetic rats increases endoneural ATPase activity in rat. Because the defect in Na^+^,K^+^-ATPase activity is also probably involved in the development of diabetic nephropathy and cardiomyopathy, physiological C-peptide infusion could be beneficial for the prevention of diabetic complications.


*Experimental Diab. Res.*, 5:37–50, 2004
Copyright © Taylor and Francis Inc.
ISSN: 1543-8600 print / 1543-8619 online
DOI: 10.1080/15438600490424514

Taylor & Francis
Taylor & Francis Group

### C-peptide, $Na^+$,$K^+$-ATPase, and Diabetes

**P. Vague, T. C. Coste, M. F. Jannot, D. Raccah, and M. Tsimaratos**

*Departement de Nutrition-Endocrinologie-Maladies Métaboliques, Marseille, France*

**$Na^+$,$K^+$-ATPase is an ubiquitous membrane enzyme that allows the extrusion of three sodium ions from the cell and two potassium ions from the extracellular fluid. Its activity is decreased in many tissues of streptozotocin-induced diabetic animals. This impairment could be at least partly responsible for the development of diabetic complications. $Na^+$,$K^+$-ATPase activity is decreased in the red blood cell membranes of type 1 diabetic individuals, irrespective of the degree of diabetic control. It is less impaired or even normal in those of type 2 diabetic patients. The authors have shown that in the red blood cells of type 2 diabetic patients, $Na^+$,$K^+$-ATPase activity was strongly related to blood C-peptide levels in non–insulin-treated patients (in whom C-peptide concentration reflects that of insulin) as well as in insulin-treated patients. Furthermore, a gene-environment relationship has been observed. The alpha-1 isoform of the enzyme predominant in red blood cells and nerve tissue is encoded by the *ATP1A1* gene. A polymorphism in the intron 1 of this gene is associated with lower enzyme activity in patients with C-peptide deficiency either with type 1 or type 2 diabetes, but not in normal individuals. There are several lines of evidence for a low C-peptide level being responsible for low $Na^+$,$K^+$-ATPase activity in the red blood cells. Short-term C-peptide infusion to type 1 diabetic patients restores normal $Na^+$,$K^+$-ATPase activity. Islet transplantation, which restores endogenous C-peptide secretion, enhances $Na^+$,$K^+$-ATPase activity proportionally to the rise in C-peptide. This C-peptide effect is not indirect. In fact, incubation of diabetic red blood cells with C-peptide at physiological concentration leads to an increase of $Na^+$,$K^+$-ATPase activity. In isolated proximal tubules of rats or in the medullary thick ascending limb of the kidney, C-peptide stimulates in a dose-dependent manner $Na^+$,$K^+$-ATPase activity. This impairment in $Na^+$,$K^+$-ATPase activity, mainly secondary to the lack of C-peptide, plays probably a role in the development of diabetic complications. Arguments have been developed showing that the diabetes-induced decrease in $Na^+$,$K^+$-ATPase activity compromises microvascular blood flow by two mechanisms: by affecting microvascular regulation and by decreasing red blood cell deformability, which leads to an increase in blood viscosity. C-peptide infusion restores red blood cell deformability and microvascular blood flow concomitantly with $Na^+$,$K^+$-ATPase activity. The defect in ATPase is strongly related to diabetic neuropathy. Patients with neuropathy have lower ATPase activity than those without. The diabetes-induced impairment in $Na^+$,$K^+$-ATPase activity is identical in red blood cells and neural tissue. Red blood cell ATPase activity is related to nerve conduction velocity in the peroneal and the tibial nerve of diabetic patients. C-peptide infusion to diabetic rats increases endoneural ATPase activity in rat. Because the defect in $Na^+$,$K^+$-ATPase activity is also probably involved in the development of diabetic nephropathy and cardiomyopathy, physiological C-peptide infusion could be beneficial for the prevention of diabetic complications.**

**Keywords** C-Peptide; Diabetes; $Na^+$,$K^+$-ATPase

Recently, several investigators have shown that C-peptide administration exercised some beneficial effects in humans or animal affected by insulinopenic diabetes and lacking both C-peptide and insulin. Among these effects, a stimulation of $Na^+$,$K^+$-ATPase activity in various tissues has been described.

It is known that $Na^+$,$K^+$-ATPase activity is impaired in the cell membrane of many tissues obtained from diabetic individuals or animals and this defect may play a role in the development of the complication of diabetes. This review will deal with the diabetes-induced alteration of $Na^+$,$K^+$-ATPase activity, its relationship with C-peptide level, the effect of C-peptide administration on $Na^+$,$K^+$-ATPase activity in diabetes, and the

Received 12 March 2003; accepted 1 June 2003.
Address correspondence to Philippe Vague, MD, PhD, Département de Nutrition-Endocrinologie-Maladies Métaboliques, CHU Timone, Boulevard Jean Moulin, 13385 Marseille Cedex 05, France. E-mail: philippe.vague@ap-hm.fr possible relevance of these findings to the comprehension of, and the therapeutic approach to prevent or treat, the long-term complications of diabetes.

### THE SODIUM-POTASSIUM ATPase

### Introduction

The sodium-potassium adenosine triphosphatase ($Na^+$,$K^+$-ATPase; sodium pump; EC 3.6.1.37) is an ubiquitous membrane-associated protein complex that is expressed in most eukaryotic cells. The "pump" transduces energy from the intracellular hydrolysis of adenosine triphosphate (ATP) to the active countertransport of sodium and potassium across the cell membrane (Clausen et al., 1991). The $Na^+$,$K^+$-ATPase contains 1 principal catalytic subunit, designated $\alpha$ and 1 sugar-rich auxiliary subunit, designated $\beta$. There is an associated subunit $\gamma$ present only in some tissues, the 3 subunits occurring in a 1:1:1 ratio (Forbush et al., 1978; Jorgensen, 1982).

The $\alpha$ subunit has a molecular mass of about 110 kDa with 10 transmembrane segments. Four distinct isoforms have been identified. The differences of amino acid sequences among the isoforms are minor. They are each coded by a different gene, some of them located on different chromosomes (Sweadner, 1989; Mercer, 1993). The various isoforms differ primarily in their tissue distribution, $\alpha 1$ predominating in several tissues, including kidney, nerves, and lung; $\alpha 2$ in skeletal muscle and heart; $\alpha 3$ in the brain; and $\alpha 4$, which is apparently localized to testis and specifically to spermatozoa (Kaplan, 1985). The $\alpha$ subunit carries the catalytic function of the enzyme, and this is reflected in its possession of several binding and functional domains.

The $\beta$ subunit has a molecular mass of about 55 kDa, with a single membrane crossing. Three isoforms have been identified. As $\alpha$ isoforms, $\beta$ isoforms have a tissue-specific distribution, $\beta 1$ is ubiquitous, $\beta 2$ is expressed in skeletal muscle and heart, and $\beta 3$ in testis and central nervous system (Kotyk and Amler, 1995). It is clear that an essential role for $\beta$ subunit is in the delivery and the appropriate insertion of $\alpha$ subunit in the membrane (McDonough et al., 1990). In recent years, a variety of studies suggest that the $\beta$ subunit may be more intimately involved in the mechanism of active transport and may be a regulatory subunit (Kaplan, 1985; Geering, 2001).

The $\gamma$ subunit is a hydrophobic and a single-membrane-crossing protein of molecular mass about 12 kDa. Although very little is known about its function, it does appear to be obligatorily associated with the $\alpha\beta$ complex (Therien et al., 1997).

$Na^+$,$K^+$-ATPase couples the energy released in the intracellular hydrolysis of ATP to the export of three intracellular sodium ions and the import of two extracellular potassium ions. The continuous operation of this macromolecular machine ensures the generation and maintenance of concentration gradients of sodium and potassium across the cell membrane. This electrochemical gradient provides energy for the membrane transport of metabolites, nutrients, and ions. This electrochemical gradient is essential also for regulation of cell volume and intracellular pH and for the action potential of muscle and nerve (Therien et al., 1997). This enzyme is responsible of about 15% to 20% of resting energetic expense in whole organism (Clausen et al., 1991).

Because several cellular transport systems are coupled to the movement of sodium and, therefore, to the function of $Na^+$,$K^+$-ATPase, this enzyme is the target of multiple regulatory mechanisms activated in response to changing cellular requirements. The requirement for modulators of the $Na^+$,$K^+$-ATPase is likely to be greatest in tissues in which perturbations of the intracellular alkali cation concentration underlie their specialized functions. Prime examples are the changes in enzyme activity that occur in response to physiological stimuli such as nerve impulse propagation and exercise (Therien et al., 1997). In general, regulation of $Na^+$,$K^+$-ATPase is brought about by increased message transcription, increased recruitment of heterodimers to the cell membrane, alterations of heterodimers trafficking, and half-life in the cell membrane, and by direct regulation of enzymes in the cell membrane. Direct regulation of the cell membrane enzymes results from phosphorylation and dephosphorylation by protein kinases and protein phosphatases. Thus, depending on the tissue, activation of protein kinases can induce an increase or decrease in sodium pump activity (Ewart and Klip, 1995). Moreover, sodium pump regulation seems to be tissue but also isoform specific (Feraille and Doucet, 2001).

Firstly, the simplest and most straightforward determinants of pump activity are the concentrations of substrates, i.e., sodium, potassium, and ATP. Some hormones appear to alter $Na^+$,$K^+$-ATPase activity by changing its apparent affinity for sodium or by increasing the sodium influx. The ATP concentration is generally saturating for the enzyme in most cells. However, in some tissues and under certain conditions, ATP levels may fall to subsaturating levels, such as in kidney medulla, which functions under near anoxic conditions (Brezis and Rosen, 1995).

Secondly, endogenous cardiotonic steroids such as ouabain inhibits specifically the sodium pump, whereas interactions of the pump with components of the cytoskeleton allows the correct processing and targeting of sodium pumps to the appropriate membrane compartment (Therien et al., 1997).

Thirdly, $Na^+$,$K^+$-ATPase is a transmembranous enzyme and many reports have focused on the role of membrane lipids. In general, lipids that promote bilayer formation of physiological thickness and increased fluidity tend to support optimal $Na^+,K^+$-ATPase activity, as do negatively charged lipids such as phosphatidylserine and phosphatidylglycerol (Kimelberg & Papahadjopoulos, 1972; Kimelberg, 1975; Johannson et al., 1981). The effects of cholesterol on enzyme activity are often also related to membrane fluidity (Giraud et al., 1981). Free fatty acids present in the membranes or as the products of phospholipases and eicosanoids tend to have various regulatory effects on $Na^+,K^+$-ATPase activity.

Lastly, the enzyme activity is subjected to both short- and long-term regulation by several hormones. Short-term regulation involves generally direct effects on the kinetic behavior of the enzyme or translocation of sodium pumps between intracellular stores and the plasma membrane. Long-term regulation induces de novo $Na^+,K^+$-ATPase synthesis or degradation. Corticosteroids and particularly aldosterone sustains a long-term increase in expression of sodium pumps, whereas catecholamines have various affects on pump activity, with an inhibitory effect of dopamine and a stimulating effect of epinephrine and norepinephrine (Therien et al., 1997). Insulin mainly stimulates the $Na^+,K^+$-ATPase activity by increasing the translocation of sodium pumps from intracellular stores to the cell surface, the cytoplasmic sodium content, and also the apparent affinity of the enzyme for sodium (Sweeney and Klip, 1998). Recently, C-peptide has been found to stimulate the $Na^+,K^+$-ATPase activity in renal tubular from control rat (Ohtomo et al., 1996) and in sciatic nerve from diabetic rat (Ido et al., 1997). In human diabetes, the decrease of availability of both insulin and C-peptide could change the regulatory equilibrium of $Na^+,K^+$-ATPase activity in favor of a decrease.

The human genome appears to contain at least five distinct $\alpha$ subunit and related genes, each 20 to 25 kb in length. $\alpha 1$, $\alpha 2$, and $\alpha 4$ are coded by *ATP1A1*, *ATP1A2*, and *ATP1AL2* genes respectively located on chromosome 1, whereas $\alpha 3$ is coded by *ATP1A3* gene located on chromosome 19. There is also a related gene, *ATP1AL1*, located on chromosome 13, with no transcription of a mRNA because of a lack of functional promoter region (Yang-Feng et al., 1988; Modyanov et al., 1991; Shamraj and Lingrel, 1994). Some polymorphisms have been identified on $\alpha$ genes, and particularly, on *ATP1A1*, which presents a restriction polymorphism with Bgl II enzyme (Shull et al., 1990).

The multiple $\beta$ isoforms are products of separate genes, about 8 kb in length, which are dispersed in the human genome. $\beta 1$, $\beta 2$, and $\beta 3$ are coded by *ATP1B1*, *ATP1B2*, and *ATP1B3*, respectively, and located on chromosomes 1, 17, and 9 (Avila et al., 1998; Mobasheri et al., 2000). A related gene, *ATP1BL1*, has been identified on chromosome 4 but seems to be not expressed (Yang-Feng et al., 1988). In parallel with $\alpha$ subunits, some polymorphisms have been identified too on $\beta$ genes (Shull et al., 1990).

### Diabetes-Induced Alterations in $Na^+,K^+$-ATPase Activity

Diabetes has a marked effect on the metabolism of a variety of tissues and because the $Na^+,K^+$-ATPase is critical for the membrane potential and many transports, a change in its activity in diabetes would have profound consequence in these tissues. Streptozotocin- and alloxan-treated or genetically susceptible diabetic rodents are animal models used to assess metabolic and physiological changes induced by insulin-dependent diabetes. Among the diabetes-induced metabolic changes, disturbances of $Na^+,K^+$-ATPase activity have been widely reported (Sima and Sugimoto, 1999). Das and colleagues (1976) first described a decrease of this enzyme activity in sciatic nerve of diabetic rat, whereas an increase in enzyme activity was found in mucosa of the small intestine of diabetic rat (Gnanaprakasam and Srivastava, 1978). These two examples illustrate the different effect of diabetes on $Na^+/K^+$-ATPase depending on the tissues. Tissues can be classified in three groups, one principal group in which diabetes induces a decrease in $Na^+,K^+$-ATPase activity, including sciatic nerve, lens, heart, and erythrocyte; another group in which diabetes causes an increase in enzyme activity, including mucosa of the small intestine; and one group with a $Na^+,K^+$-ATPase activity unchanged or where it exists differences between studies. The last group includes tissues such as brain and kidney. All the tissues variations in $Na^+,K^+$-ATPase activity in diabetic rat are summarized in Table 1.

Various mechanisms have been suggested to explain the decrease in $Na^+,K^+$-ATPase activity: a depletion of the intracellular pool of *myo*-inositol, an increased flux through the aldose reductase pathway, and an alteration in protein kinase C (PKC) activity (Greene et al., 1987). Others diabetes-induced metabolic changes can also down-regulate the enzyme activity, including the increase in oxidative stress, the formation of advanced glycation products, the nerve growth factor metabolism (Sima and Sugimoto, 1999), and the disturbance in essential fatty acid metabolism leading to an abnormal $\omega 6/\omega 3$ ratio in red blood cell membrane (Djemli-Shipkolye et al., 2003). Moreover, insulin and C-peptide deficiencies can alter the long-term regulation of enzyme units expressed at the cell surface. Indeed, some studies have found significant decreases in mRNA levels of one to four subunits in aorta and left ventricle as soon as 14 days after diabetes induction (Ohara et al., 1991; Ver et al., 1997). Interestingly, Nishida and colleagues (1992) have shown that in skeletal muscle of diabetic rat, mRNA level of $\alpha 1$ was unchanged, whereas mRNA level of $\alpha 2$ was increased 14 days after diabetes induction. However, $Na^+,K^+$-ATPase activity was diminished because of a significant decrease in mRNA level of the $\beta 1$ isoform (Nishida et al., 1992). The last point shows that overexpression of $\alpha$ isoforms cannot be connected with an increase in enzyme activity; $\beta$ isoforms are necessary to form

**TABLE 1**
Variations in $Na^+,K^+$-ATPase activity in different tissues of insulinopenic diabetic rat

| Tissue | $Na^+,K^+$-ATPase activity | Reference |
|---|---|---|
| Brain, nervous system, and glands | | |
| Brain | ⇔ | Mayanil et al., 1982 |
| Brain | ⇓ | Ver et al., 1995a |
| Dorsal root ganglia | ⇓ | Green et al., 1985 |
| Hippocampus, cerebral cortex | ⇓ | Leong and Leung, 1991 |
| Thalamus, hypothalamus, brain stem | ⇓ | Leong and Leung, 1991 |
| Striatum, cerebellum | ⇔ | Leong and Leung, 1991 |
| Superior cervical ganglion | ⇓ | Greene and Mackway, 1986 |
| Lens | ⇓ | Ahmad et al., 1985 |
| Retinal pigmented epithelium | ⇓ | McGregor et al., 1986 |
| Sciatic nerve | ⇓ | Das et al., 1976 |
| Vagal nerve | ⇓ | Nowak et al., 1995 |
| Heart | | |
| Heart sarcolemma | ⇓ | Pierce and Dhalla, 1983 |
| Heart ventricular muscle | ⇓ | Kjeldsen et al., 1987 |
| Intestine | | |
| Mucosa of the small intestine | ⇑ | Gnanaprakasam and Srivastava, 1978 |
| Mucosa basolateral membrane | ⇑ | Luppa and Muller, 1986 |
| Mucosa brush border region | ⇔ | Luppa and Muller, 1986 |
| Kidney | | |
| Whole kidney | ⇑ | Clark et al., 1983 |
| Collecting tubule | ⇑ | Trinh-Trang-Tan et al., 1985 |
| Cortical thick ascending limb | ⇔ | Trinh-Trang-Tan et al., 1985 |
| Glomeruli (isolated) | ⇓ | Cohen et al., 1985 |
| Medullary thick ascending limb | ⇔ | Trinh-Trang-Tan et al., 1985 |
| Medullary thick ascending limb | ⇓ | Tsimaratos et al., 2001 |
| Renal medulla and cortex | ⇑ | Wald and Popovtzer, 1984 |
| Renal tubule | ⇑ | Ku et al., 1986 |
| Others tissues | | |
| Aorta | ⇓ | Ohara et al., 1991 |
| Erythrocyte | ⇓ | Agarwal et al., 1985 |
| Hepatocyte | ⇓ | Carnovale et al., 1991 |
| Liver | ⇑ | Sennoune et al., 2000 |
| Skeletal muscle | ⇓ | Kjeldsen et al., 1987 |

⇑ increased; ⇓ decreased; ⇔ unchanged.

an active complex. It will be shown below that C-peptide has short-term effect or $Na^+,K^+$-ATPase activity by modifying its phosphorylation status.

Contrary to the down-regulation mechanisms, the mechanisms implicated in the $Na^+,K^+$-ATPase increase in some tissues of diabetic rat seems to be related to variations on mRNA levels of $Na^+,K^+$-ATPase isoforms. For example, in small intestine, an increase in $Na^+,K^+$-ATPase activity is correlated with an increase in mRNA levels of $\alpha 1$ and $\beta 1$ isoforms (Fedorak et al., 1991; Barada et al., 1994). In kidney, Ver and colleagues (1995b) have shown that mRNA levels of $\alpha 1$ and $\beta 1$ isoforms increased in medulla but not in cortex, whereas there was a highly significant linear correlation between $Na^+,K^+$-ATPase activity and mRNA level of $\alpha 1$ isoforms in all the nephron segments in the study of Scherzer and Popovtzer (2002). The discrepancies in enzyme activity observed in the diabetic kidney can be attributed to time-dependent changes in $Na^+,K^+$-ATPase activity and expression. Early renal hypertrophy in diabetic rat enhances $Na^+,K^+$-ATPase activity in several nephron segments secondary to a recruitment of enzyme stores and an increase in the amount of $\alpha 1$ and $\beta 1$ subunits. Long-term diabetes (12 weeks and more) can decrease the $Na^+,K^+$-ATPase activity, with a reduction in $\alpha 1$ and $\beta 1$ isoforms abundance, particularly in medullary thick ascending limb (Tsimaratos et al., 2001).

### $Na^+,K^+$-ATPase Abnormalities in Human Diabetes

In human individuals, the vast majority of the studies have been done on erythrocyte, except for a few on nerve biopsies (Scarpini et al., 1993). The red blood cell (RBC) $Na^+,K^+$-ATPase activity reflects that of the sciatic nerve (Raccah et al., 1996b). As shown in Table 1, diabetes induces a decrease in the enzyme activity in almost all the tissues as well as in erythrocytes. Therefore, it seems reasonable to suggest that $Na^+,K^+$-ATPase activity data obtained from RBCs may be extrapolated to the enzyme status in other tissues, specially in nerve tissue.

Earlier studies (Finotti and Verbaro, 1987; Rahmani-Jourdheuil et al., 1987) have shown that in patients affected by type 1 diabetes, RBC $Na^+,K^+$-ATPase activity was decreased, as it is in diabetic animals. In type 1 diabetic individuals, RBC $Na^+,K^+$-ATPase activity is on the average 30% lower that in appropriate control individuals (Figure 1). Various approaches may be used to estimate the enzyme function, which may be done by measuring ATP hydrolysis by red blood cell membrane (Raccah et al., 1992), sodium efflux (Finotti and Verbaro, 1987), rubidium influx (Kimelberg and Mayhew, 1975), or energy expenditure by a microcaloric method (Issautier et al., 1994) in living RBCs. Comparable results have been obtained with all these techniques, mainly a 30% decrease in enzyme activity in RBCs from diabetic patients compared to control individuals.

The impaired enzyme activity does not appear linked to a lower number of enzyme units in the RBC membrane as estimated by [$^3$H]ouabain binding (Raccah et al., 1996a). No correlation was observed between RBC $Na^+,K^+$-ATPase activity and the degree of metabolic control estimated by actual blood glucose or $HbA_{1c}$ nor with cholesterol or triglyceride levels, insulin requirement, or body mass index (Raccah et al., 1996b). Therefore, hyperglycemia does not appear directly responsible for the changes in enzyme activity. As it will be described below, C-peptide deficiency appears strongly implicated.

Another argument to exclude a direct responsibility of hyperglycemia is that in type 2 diabetic individuals, RBC $Na^+,K^+$-ATPase activity is much less impaired, as shown on Figure 1. In fact, it appears in the normal range in the groups of individuals treated by diet alone or oral agents, and impaired in the insulin-treated group only.

### Genetic Control of $Na^+,K^+$-ATPase Activity

Several findings suggest that $Na^+,K^+$-ATPase activity is under genetic control. It is lower in men (Lasker et al., 1985). Strong familial influence and ethnic differences have been noted, with lower levels being observed in blacks, Asians, and Jewish subjects in comparison to Scandinavians (Beutler et al., 1983; Lasker et al., 1985). Having observed that type 1 diabetic individuals of North African ancestry present with an increased prevalence of polyneuropathy than their European counterpart

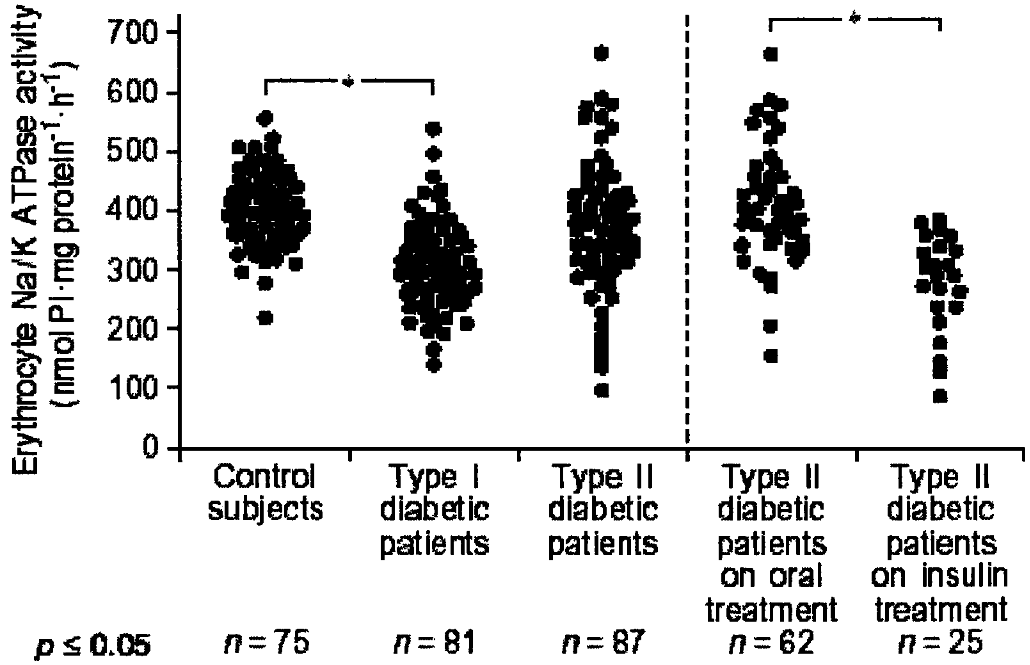

**FIGURE 1**

Erythrocyte $Na^+,K^+$-ATPase activity in the different groups of control subjects and type 1 and type 2 diabetic patients. From Dufayet de la Tour et al., 1998.

(Vague et al., 1988), and suspecting the role of a decreased $Na^+,K^+$-ATPase activity in this complication, we observed that RBC $Na^+,K^+$-ATPase activity was lower in subjects of North African ancestry, either healthy or diabetic, compared to their appropriate European controls. This phenomenon appears to be related to a decrease in a number of enzyme units in the RBC membrane, as estimated by a lower number of ouabain binding sites (Vague et al., 1997b).

As mentioned above, a polymorphism with a variant allele frequency around 0.1 has been described in the intron 1 of the *ATPA1* gene encoding for the $\alpha 1$ isoform, major isoform of the $\alpha$ subunit, which is the only one present in red blood cells. RBC $Na^+,K^+$-ATPase activity does not differ between nondiabetic individuals bearing or not the variant allele. However, among diabetic subjects, RBC ATPase activity is even lower in subjects with the variant allele (Jannot et al., 2002). The presence of this allele could confer a genetic predisposition to some diabetic complications (Vague et al., 1997a).

### RELATIONSHIP BETWEEN $Na^+,K^+$-ATPase AND C-PEPTIDE IN DIABETES

The diabetes-induced impairment in $Na^+,K^+$-ATPase activity could be related to hyperglycemia via an increased glycosylation of its $\beta$ subunit involved in the maturation of the enzyme and its localization and stabilization in the plasma membrane (Eakle et al., 1994). The defect in *myo*-inositol metabolism leading to altered lipid metabolism in the membrane and by the way decreased $Na^+,K^+$-ATPase activity could also be involved (Yorek et al., 1988). However, a direct action of hyperglycemia on this enzyme activity has never been confirmed.

In type 1 diabetic patients, RBC $Na^+,K^+$-ATPase activity is not related to actual plasma glucose, nor $HbA_{1c}$ value, plasma triglyceride, or total cholesterol (Raccah et al., 1996b). More in type 2 diabetes, this enzyme activity is much less impaired than in type 1 diabetes in spite of similar $HbA_{1c}$ values (Jannot et al., 2002). The same has been observed for the sciatic nerve $Na^+,K^+$-ATPase activity in type 2 diabetic BBZDR rats compared to type 1 diabetic BB rats (Sima et al., 2000).

Therefore, hyperglycemia per se does not seem to be responsible for the impairment in $Na^+,K^+$-ATPase activity.

Among type 2 diabetic patients, the only parameter found to be independently associated with RBC $Na^+,K^+$-ATPase activity was the plasma C-peptide concentration (Table 2, Figure 2; Dufayet de la Tour et al., 1998). Because C-peptide is cosecreted with insulin and the plasma C-peptide level reflects that of insulin, a relation between C-peptide and $Na^+,K^+$-ATPase activity could reflect in fact a relationship with insulin. However, Figure 2 shows that the relationship between C-peptide and $Na^+,K^+$-ATPase activity in type 2 diabetic patients persists among insulin-treated patients and is even stronger in this subgroup ($r = .64$ versus .41 for all groups), establishing clearly that RBC $Na^+,K^+$-ATPase activity is related to C-peptide levels and not to insulin levels.

**TABLE 2**
Stepwise regression analysis of the correlation between erythrocyte $Na^+,K^+$-ATPase activity and the different parameters in type 2 diabetic patients

| | Correlation coefficient | $F$ | $P$ value |
|---|---|---|---|
| C-peptide | .48 | 7.7 | .002 |
| Age | −.2 | 3.4 | NS |
| Diabetes duration | −.17 | 1.9 | NS |
| Type of treatment[a] | .09 | 0.04 | NS |
| $HbA_{1c}$ | −.089 | 0.04 | NS |
| BMI | −.087 | 0.03 | NS |

*Note*. From Dufayet de la Tour et al., 1998.
[a]Insulin or oral hypoglycemic agents.

The presence of a variant allele of the *ATP1A1* gene encoding for the $\alpha 1$ isoform of the $Na^+,K^+$-ATPase is associated with lower $Na^+,K^+$-ATPase activity in type 1 diabetic patients but not in control subjects (Vague et al., 1997a), which means that the presence of the variant allele makes ATPase activity more sensitive to the deleterious effect of diabetes, this effect being probably secondary to the lack of C-peptide. And, in fact, we have observed that in type 2 diabetic individuals, ATPase activity is dependent both on the presence of the variant allele and C-peptide levels (Jannot et al., 2002).

Its seems clearly demonstrated that low $Na^+,K^+$-ATPase activity in RBCs and sciatic nerves of diabetic individuals is related to low C-peptide levels. Is this relationship a causal one?

### EFFECT OF C-PEPTIDE ADMINISTRATION ON $Na^+,K^+$-ATPase ACTIVITY

### In Vivo Effect

Patients affected by type 1 diabetes and who were C-peptide negative received after a fasting period of about 5 hours a continuous infusion of either C-peptide or placebo. C-peptide was infused at a concentration of 3 $pmol/min^{-1}kg^{-1}$ for the first 60 minutes, then at a concentration of 10 $pmol^{-1}kg^{-1}$ for another 6 minutes (Forst et al., 2000). C-peptide levels reached a maximum of $1.2 \pm 0$ $nmol{\cdot}L^{-1}$ at the end of the first infusion period and $3.5 \pm 0.3$ $nmol{\cdot}L^{-1}$ after 120 minutes. These values are in the physiological fasting and postprandial range, respectively.

Levels of erythrocyte $Na^+,K^+$-ATPase activity increased during the C-peptide infusion by $83.3 \pm 34.1$ nmol $P_i$ $h^{-1}$ per mg protein during the low infusion rate and by $146 \pm 6.9$ nmol $P_i$ $h^{-1}$ per mg protein during the high infusion rate. The

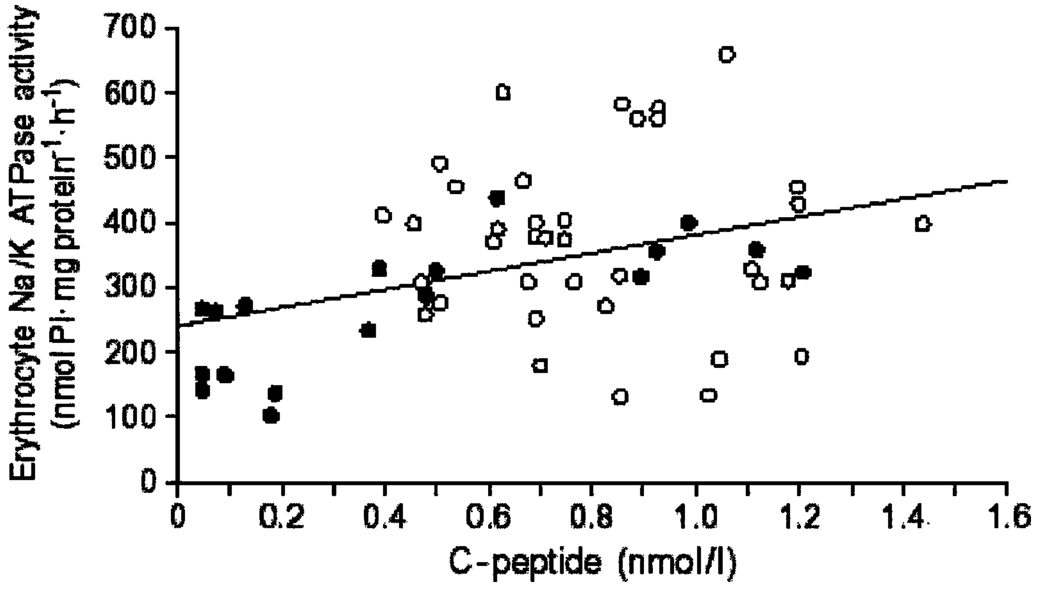

**FIGURE 2**

Correlation between fasting C-peptide and erythrocyte $Na^+$,$K^+$-ATPase activity among the type 2 diabetic patients. ○ Type 2 diabetic patients on oral treatment. ● Type 2 diabetic patients on insulin treatment.

percentage increase in enzyme activity during the two periods were respectively 62% ± 14% and 113% ± 20%. Although $Na^+$,$K^+$-ATPase activity increased slightly during saline infusion, the changes were not significant.

### In Vitro Effect

This effect of C-peptide on erythrocyte $Na^+$,$K^+$-ATPase activity in vivo could be direct or indirect. It is in fact a direct one. Erythrocytes obtained from C-peptide–negative diabetic patients were resuspended in saline to eliminate leukocytes and platelets and filtered trough a cellulose microcrystalline column. They were then incubated in a water bath at 37°C with C-peptide (6 nmol/L) for 10 minutes. $Na^+$,$K^+$-ATPase activity was significantly increased in the presence of C-peptide (363 ± 30 versus 255 ± 22 nmol/$P_i$/mg protein/h; $P = .002$ n = 14) (Djemli-Shipkolye et al., 2000). The values, which were lower than normal, in the "diabetic" range, returned to the normal range.

Renal tubular cells reabsorb a huge amount of sodium in part through $Na^+$,$K^+$-ATPase activity. Accordingly, they are a rich source of $Na^+$,$K^+$-ATPase. The basic function of the $Na^+$,$K^+$-ATPase is to maintain high sodium and potassium gradients across the plasma membrane of animal cells (for review, see Feraille and Doucet, 2001). Indeed, in rat proximal convoluted tubule (Ohtomo et al., 1996, 1998) and in medullary thick ascending limb (MTAL) (Tsimaratos et al., 2003), C-peptide induced a dose-dependent stimulation of $Na^+$,$K^+$-ATPase activity.

These results were also reported with ouabain-sensitive uptake of rubidium-86, a marker of $Na^+$,$K^+$-ATPase activity, in primary culture of human renal tubular cells (Johansson et al., 2002) and in native rat MTAL tubules (Tsimaratos et al., 2003) after exposure to autologous C-peptide.

### MECHANISMS BY WHICH C-PEPTIDE STIMULATES $Na^+$,$K^+$-ATPase ACTIVITY

Almost of all these studies were performed on renal tubular cells in which $Na^+$,$K^+$-ATPase is particularly abundant and deeply involved in the main physiological function of these cells, i.e., the reabsorption of sodium.

### Signal Transduction Pathway

The signal transduction pathway is thought to involve a G-protein–coupled receptor because $Na^+$,$K^+$-ATPase activity was abolished when renal tubules were incubated with pertussis toxin (Ohtomo et al., 1996). In the same experiment and in others, there was evidence that the effect observed was dependent on a rise of intracellular calcium (Ohtomo et al., 1996; Shafqat et al., 2002). Accurately, the C-peptide–induced increase of intracellular calcium is mediated by a calcium influx because EGTA abolished its effect (Ohtomo et al., 1996). Furthermore, pertussis toxin inhibited both C-peptide–induced $Na^+$,$K^+$-ATPase activation and an increase in intracellular calcium concentration (Agarwal et al., 1985), suggesting a link between these two processes. Interestingly, C-peptide stimulated specifically the classical PKC-$\alpha$, calcium-dependent mediator of $Na^+$,$K^+$-ATPase phosphorylation in tubular cells (Tsimaratos et al., 2003). PKC-$\delta$, -$\varepsilon$, -$\varsigma$, which are also expressed in this nephron segment, were not affected by C-peptide stimulation.

Because C-peptide specifically stimulated $Na^+$,$K^+$-ATPase and PKC-$\alpha$ translocation in MTAL cells, and because PKC phosphorylates tubular $Na^+$,$K^+$-ATPase (Feraille et al., 1995), the effect of GF109203X, a specific inhibitor of PKCs, was examined. GF109203X completely abolished C-peptide–induced $Na^+$,$K^+$-ATPase activity together with PKC translocation.

In addition to PKC activation, calcium-dependent mitogen-associated protein kinase activation (MAPK) ERK 1 and 2 phosphorylation, abolished by pertussis toxin, was described in cultured cells of mouse embryonic fibroblast cell line (Swiss 3T3) (Kitamura et al., 2001), and in human tubular cells (Johansson et al., 2002). The latter argues for a possible recruitment of tissue-specific intracellular effectors of $Na^+$,$K^+$-ATPase activation, rather than direct effect of PKC.

The contribution of adenyl cyclase activation to C-peptide effect was observed in cultured cells (Kitamura et al., 2001), but not in native MTAL cells, because physiological amounts of C-peptide failed to increase the cellular cAMP release through adenyl cyclase activity (Tsimaratos et al., 2003). These results were consistent with the lack of increase of $Na^+$,$K^+$-ATPase amount to membrane in response to C-peptide in MTAL (Tsimaratos et al., 2003), because in this segment the protein kinase A (PKA/cAMP) pathway results in recruitment of active $Na^+$,$K^+$-ATPase units (for review, see Feraille and Doucet, 2001).

### $Na^+$,$K^+$-ATPase Phosphorylation

There is now evidence that C-peptide effect on $Na^+$,$K^+$-ATPase activity is mediated in part by phosphorylation of the catalytic $\alpha$ subunit.

Incubation of native MTAL tubules with physiological amounts of C-peptide resulted in phosphorylation of $Na^+$,$K^+$-ATPase catalytic subunit, which was abolished after GF109203X preincubation of tubules, indicating that the effect of C-peptide on $Na^+$,$K^+$-ATPase activity was dependent on PKC-$\alpha$–dependent phosphorylation of the catalytic subunit (Tsimaratos et al., 2003).

### C-peptide Receptor

The stimulatory effect of C-peptide on $Na^+$,$K^+$-ATPase was concentration dependent, and abolished by pertussis toxin (Ohtomo et al., 1996; Tsimaratos et al., 2003). The kinetics studies showed that physiological amounts of C-peptide increased $Na^+$,$K^+$-ATPase activity after 5 minutes and that this effect reached a plateau from 15 to 60 minutes (Tsimaratos et al., 2003).

Therefore, the mechanism involved in C-peptide effect was thought to be secondary to a specific linkage of C-peptide to renal cells. Because pertussis toxin inhibited C-peptide effect in various cells (Ohtomo et al., 1996; Rigler et al., 1999; Johansson et al., 2002), the receptor was suspected to be a G-protein–type receptor, resulting in calcium influx and PKC and MAPK activation.

Altogether, these arguments are in favor of a specific hormonal effect in renal cells where C-peptide stimulates $Na^+$,$K^+$-ATPase activity (Ohtomo et al., 1996; Johansson et al., 2002; Tsimaratos et al., 2003), PKC-$\delta$ (Tsimaratos et al., 2003), calcium influx (Shafqat et al., 2002), and activation of MAPK (Kitamura et al., 2001; Johansson et al., 2002), but not PKA (Tsimaratos et al., 2003). Furthermore, pertussis toxin inhibits most of these effects, suggesting that C-peptide binds to transmembrane G-protein–coupled receptors with the $\alpha$-subunit of the $G_i/G_0$ subtype. Activation of PKC and phosphoinositide 3-kinase (PI3K) is likely to be involved in C-peptide–induced phosphorylation of MAPKs (Kitamura et al., 2001). However, C-peptide specifically increased the amounts of cell membrane–associated PKC-$\alpha$ (an index of its activation; Karim et al., 1995), whereas the amounts of membrane-associated PKC-$\delta$ and PKC-$\varsigma$ isoforms remained unaltered (Tsimaratos et al., 2003). The specific PKC activation (Tsimaratos et al., 2003) is consistent with the effect of C-peptide on $Na^+$,$K^+$-ATPase activity, depending on the pertussis toxin–sensitive G-protein, and on an increase in intracellular calcium concentration (Ohtomo et al., 1996). In addition, GF109203X abolished $Na^+$,$K^+$-ATPase activation and phosphorylation induced by C-peptide when used at concentrations that inhibit classical and novel PKCs but not atypical PKCs (Martiny-Baron et al., 1993). Together with its saturable effect, these findings strongly suggest that C-peptide acts through a G-protein–coupled receptor linked to the classical PKC-$\alpha$ pathway in rat MTAL.

### C-PEPTIDE, $Na^+$,$K^+$-ATPase AND THE COMPLICATIONS OF DIABETES

There are several lines of evidence suggesting that the ability of C-peptide substitution to prevent and maybe to reverse some complications of diabetes is mediated by the restoration of $Na^+$,$K^+$-ATPase activity.

Indeed, abnormalities in this enzyme activity appear implicated in a variable extent in the pathogenesis of many complications of diabetes.

### Erythrocyte Dysfunction

It is well known that the properties of diabetic erythrocytes are abnormal (Juhan et al., 1982; Finotti and Verbaro, 1987). These abnormalities include decreased deformability

(McMillan, 1975; Vague and Juhan, 1983) increased membrane viscosity (Baba et al., 1979) and increased erythrocyte aggregation (Schmid-Schonbein and Volger, 1976). The decreased $Na^+$,$K^+$-ATPase activity observed in the diabetic erythrocyte membrane leads to an intra cellular accumulation of sodium with subsequent accumulation of free calcium ions due to competition in transmembranous exchange (Gardner and Bennett, 1986) and may be responsible for the decreased deformability. Indeed, attenuation of $Na^+$,$K^+$-ATPase activity has been shown to correlate with decreased deformability in diabetic erythrocyte (Finotti and Verbaro, 1987). Incubation with C-peptide restores their deformability. It appears $Na^+$,$K^+$-ATPase mediated as a pretreatment by ouabain a specific $Na^+$,$K^+$-ATPase inhibitor suppresses entirely the beneficial effect of C-peptide (Kunt et al., 1999).

### Nerve Function

In experimental diabetic neuropathy, a decrease in nerve conduction velocity (NCV) has been widely reported (Sima and Sugimoto, 1999). Among the hypothetical mechanisms, the importance of a decrease in $Na^+$,$K^+$-ATPase activity has been actively debated (Greene and Lattimer, 1983; Cameron et al., 1994). A sciatic nerve decrease in this enzyme activity could alter the normal membrane axon repolarization after the depolarization induced by an action potential, and one could expect, therefore, a decrease in NCV. Wright and Nukada (1994) found significant reductions in NCV 16 weeks after diabetes induction, with trends apparent after 4 weeks. Because diabetes was induced in mature rats in this study, there was a delay before the NCV decrease became significant. Indeed, when diabetes was induced in growing rats, a significant decrease in NCV was observed as early as 2 weeks (Mizuno et al., 1999; Coppey et al., 2000). Disturbances in $Na^+$,$K^+$-ATPase activity in the sciatic nerve have been proposed to be partially but not fully responsible for the slowing of nerve conduction velocity in the diabetic rat, because in some studies, the slowing of motor NCV seems to precede the decrease in enzyme activity (Lambourne et al., 1988; Wright and Nukada, 1994). In studies from our laboratory, we always observe a partial restoration of $Na^+$,$K^+$-ATPase activity associated with either a partial (Gerbi and Maixent, 1999; Gerbi et al., 1999) or a total (Coste et al., 1999) restoration of conduction velocity in sciatic nerve of diabetic rats after various therapeutic interventions, such as gamma-linolenic acid or fish oil supplementation. In addition, Coppey and colleagues (2000) showed that the temporal decrease between these two parameters was similar, although the decrease in motor NCV became significant more rapidly (14 days) than the decrease in $Na^+$,$K^+$-ATPase activity (28 days). All these observations argue for a partial implication of a decrease in $Na^+$,$K^+$-ATPase as the cause of the diabetes-induced decrease in NCV.

Decrease $Na^+$,$K^+$-ATPase activity is associated with increased inactivation of sodium channels and intra-axonal sodium accumulation at the node, resulting in paranodal swelling (Sima and Sugimoto, 1999). C-peptide administration, which restores $Na^+$,$K^+$-ATPase activity, prevents and repairs nodal and paranodal changes in insulinopenic diabetic rats (Sima et al., 2001). In the same way, fish oil administration to streptozotocin-induced diabetic rats restores, in parallel, nerve $Na^+$,$K^+$-ATPase activity and NCV, and prevents the nerve histological damage (endoneural edema and axonal degeneration) (Gerbi et al., 1999).

Although indirect, these data support the hypothesis of a partial causative role of impaired $Na^+$,$K^+$-ATPase activity in diabetic polyneuropathy. The beneficial C-peptide effect is probably mediated by the improvement of ATPase activity together with that on nerve blood flow.

### Renal Function

The initial repercussion of diabetes on renal function are characterized by a glomerular hyperfiltration, a defect in the control of natriuresis, with a decreased responsiveness to antidiuretic hormone and later on a microalbuminuria (Zerbe et al., 1979; Wald and Popovtzer, 1984; Tucker et al., 1993; McKenna et al., 1999, 2000).

Short-term infusion of C-peptide in rats with streptozotocin-induced diabetes resulted in reduction of glomerular hyperfiltration and microalbuminuria, and regression of glomerular hypertrophy (Sjoquist et al., 1998; Samnegard et al., 2001).

In humans, treatment with C-peptide reduces both glomerular hyperfiltration and urinary albumin excretion in patients with type 1 diabetes, suggesting a specific effect on glomerular function at the early stage of diabetic nephropathy (Johansson et al., 1992, 1993, 2000).

In rat MTAL, the C-peptide effects are well documented, and insulin displays only a small stimulatory effect on $Na^+$,$K^+$-ATPase and sodium reabsorption (Mandon et al., 1993), probably due to poor expression of insulin receptors (Butlen et al., 1988). These arguments suggest strongly that in rat tubule, a physiological amount of C-peptide exerts a hormonal effect at a cellular level. The results in native tissue are consistent with a contribution of C-peptide to the multihormonal stimulation of $Na^+$,$K^+$-ATPase activity during nonfasting periods and thereby to renal sodium handling during nonfasting periods. Taken together, all these results strongly suggest that the MTAL is the physiological target of C-peptide in the kidney.

### Cardiovascular Function

Diabetes mellitus enhances the risk of morbidity and mortality from cardiovascular disease. Independently of coronary artery disease, the development of cardiomyopathy may contribute to the diabetic cardiac disease (Fein and Sonnenblick, 1985; Sonnenblick et al., 1985). Diabetic cardiomyopathy has been associated with changes in enzymatic activity in the cardiac sarcolemma with decreased $Na^+,K^+$-ATPase activity (Imanaga, 1976; Ng et al., 1993; Gerbi et al., 1997). This decreased activity averages 30%, as in other tissues affected by diabetes (Gerbi et al., 1997). One may speculate that the reduced enzyme activity impairs myocardial contractility and plays a role in a development of diabetic cardiomyopathy (Pierce and Dhalla, 1983; Schaffer, 1991). Indeed, it seems reasonable to assume that intracellular sodium and potassium homeostasis is altered. Fish oil supplementation, which restores the membrane fluidity, has been reported to lower the risk of cardiovascular disease (Weaver and Holub, 1987; Leaf and Weber, 1988). It corrects impaired heart performance in diabetic rat (Black et al., 1989) and prevents fatal ventricular fibrillation induced by ischemia (McLennan et al., 1985, 1988). It has been demonstrated that fish oil supplementation prevents and/or restores the activity of $Na^+,K^+$-ATPase in the myocardial membrane without changing its expression, probably by modifying its membrane environment (Gerbi et al., 1997). It has been recently demonstrated that a short-term 60-minute C-peptide infusion to type 1 diabetic patients improves myocardial blood flow and function (Hansen et al., 2002). It is possible that this effect is related to an improvement in the myocardium $Na^+,K^+$-ATPase activity. In the same way, it has been shown that diabetic patients who have received an islet transplant, restoring some degree of C-peptide secretion, have a better cardiovascular prognosis than the patients in whom the transplant failed, although the glycemic control was approximately the same in the two groups (La Rocca et al., 2001).

### CONCLUSIONS

An impairment of the enzyme $Na^+,K^+$-ATPase in many tissues obtained from diabetic animals as well as in humans is now well established. They are strong arguments suggesting that this impairment plays a role in the development of diabetes-associated complications.

A new aspect in the physiopathology of type 1 diabetes is that, along side the lack of insulin, C-peptide could have, although to a much lower extent, some implications. In other terms, C-peptide has a biological effect. This effect is at least in part mediated by a direct activation of $Na^+,K^+$-ATPase, more exactly by a restoration of the normal enzyme activity in the tissues of diabetic animals or humans.

If futures studies demonstrate C-peptide replacement is of substantial clinical benefit, it would probably be relatively easy to add in the insulin vial an equimolar amount of C-peptide, thus mimicking the insulin secretory granule.

### REFERENCES

Agarwal, V. R., Rastogi, A. K., Sahib, M. K., and Sagar, P. (1985) In vitro insulin action on different ATPases of erythrocyte membranes in normal and diabetic rats. *Acta Diabetol. Lat.,* **22,** 111–118.

Ahmad, S. S., Tsou, K. C., Ahmad, S. I., Rahman, M. A., and Kirmani, T. H. (1985) Studies on cataractogenesis in humans and in rats with alloxan-induced diabetes. I. Cation transport and sodium-potassium-dependent ATPase. *Ophthalmic Res.,* **17,** 1–11.

Avila, J., Alvarez de la Rosa, D., Gonzalez-Martinez, L. M., Lecuona, E., and Martin-Vasallo, P. (1998) Structure and expression of the human Na,K-ATPase beta 2-subunit gene. *Gene,* **208,** 221–227.

Baba, Y., Kai, M., Kamada, T., Setoyama, S., and Otsuji, S. (1979) Higher levels of erythrocyte membrane microviscosity in diabetes. *Diabetes,* **28,** 1138–1140.

Barada, K., Okolo, C., Field, M., and Cortas, N. (1994) Na,K-ATPase in diabetic rat small intestine. Changes at protein and mRNA levels and role of glucagon. *J. Clin. Invest.,* **93,** 2725–2731.

Beutler, E., Kuhl, W., and Sacks, P. (1983) Sodium-potassium-ATPase activity is influenced by ethnic origin and not by obesity. *N. Engl. J. Med.,* **309,** 756–760.

Black, S. C., Katz, S., and McNeill, J. H. (1989) Cardiac performance and plasma lipids of omega-3 fatty acid-treated streptozocin-induced diabetic rats. *Diabetes,* **38,** 969–974.

Brezis, M., and Rosen, S. (1995) Hypoxia of the renal medulla—its implications for disease. *N. Engl. J. Med.,* **332,** 647–655.

Butlen, D., Vadrot, S., Roseau, S., and Morel, F. (1988) Insulin receptors along the rat nephron: [125I] insulin binding in microdissected glomeruli and tubules. *Pflugers Arch.,* **412,** 604–612.

Cameron, R., Klein, L., Shyjan, A. W., Rakic, P., and Levenson, R. (1994) Neurons and astroglia express distinct subsets of $Na^+,K^+$-ATPase alpha and beta subunits. *Brain Res. Mol. Brain Res.,* **21,** 333–343.

Carnovale, C. E., Roma, M. G., Monti, J. A., and Rodriguez Garay, E. A. (1991) Studies on the mechanism of bile salt-independent bile flow impairment in streptozotocin-induced hepatotoxicity. *Toxicology,* **68,** 207–215.

Clark, D. L., Hamel, F. G., and Queener, S. F. (1983) Changes in renal phospholipid fatty acids in diabetes mellitus: correlation with changes in adenylate cyclase activity. *Lipids,* **18,** 696–705.

Clausen, T., Van Hardeveld, C., and Everts, M. E. (1991) Significance of cation transport in control of energy metabolism and thermogenesis. *Physiol. Rev.,* **71,** 733–774.

Cohen, M. P., Dasmahapatra, A., and Shapiro, E. (1985) Reduced glomerular sodium/potassium adenosine triphosphatase activity in acute streptozocin diabetes and its prevention by oral sorbinil. *Diabetes,* **34,** 1071–1074.

Coppey, L. J., Davidson, E. P., Dunlap, J. A., Lund, D. D., and Yorek, M. A. (2000) Slowing of motor nerve conduction velocity in streptozotocin-induced diabetic rats is preceded by impaired vasodilation in arterioles that overlie the sciatic nerve. *Int. J. Exp. Diabetes Res.,* **1,** 131–143.

Coste, T., Pierlovisi, M., Leonardi, J., Dufayet, D., Gerbi, A., Lafont, H., Vague, P., and Raccah, D. (1999) Beneficial effects of gammalinolenic acids supplementation on nerve conduction velocity, $Na^+,K^+$-ATPase activity, and membrane fatty acid composition in sciatic nerve of diabetic rats. *J. Nutr. Biochem.,* **10,** 411–420.

Das, P. K., Bray, G. M., Aguayo, A. J., and Rasminsky, M. (1976) Diminished ouabain-sensitive, sodium-potassium ATPase activity in sciatic nerves of rats with streptozotocin-induced diabetes. *Exp. Neurol.,* **53,** 285–288.

Djemli-Shipkolye, A., Gallice, P., Coste, T., Jannot, M. F., Tsimaratos, M., Raccah, D., and Vague, P. (2000) The effects ex vivo and in vitro of insulin and C-peptide on the $Na^{+}$,$K^{+}$-ATPase activity in red blood cell membranes of type I diabetic patients. *Metabolism,* **49,** 868–872.

Djemli-Shipkolye, A., Raccah, D., Pieroni, G., Vague, P., Coste, T. C., and Gerbi, A. (2003) Differential effect of w3 PUFA supplementations on Na,K-ATPase and Mg-ATPase activities: Possible role of the membrane w6/w3 Ratio. *J. Membr. Biol.,* **191,** 37–47.

Dufayet de la Tour, D., Raccah, D., Jannot, M. F., Coste, T., Rougerie, C., and Vague, P. (1998) Erythrocyte $Na^{+}$,$K^{+}$-ATPase activity and diabetes: Relationship with C-peptide level. *Diabetologia,* **41,** 1080–1084.

Eakle, K. A., Kabalin, M. A., Wang, S. G., and Farley, R. A. (1994) The influence of beta subunit structure on the stability of Na+,K(+)-ATPase complexes and interaction with $K^{+}$. *J. Biol. Chem.,* **269,** 6550–6557.

Ewart, H. S., and Klip, A. (1995) Hormonal regulation of the Na(+)-K(+)-ATPase: Mechanisms underlying rapid and sustained changes in pump activity. *Am. J. Physiol.,* **269,** C295–C311.

Fedorak, R. N., Cortas, N., and Field, M. (1991) Diabetes mellitus and glucagon alter ouabain-sensitive Na(+)-K(+)-ATPase in rat small intestine. *Diabetes,* **40,** 1603–1610.

Fein, F. S., and Sonnenblick, E. H. (1985) Diabetic cardiomyopathy. *Prog. Cardiovasc. Dis.,* **27,** 255–270.

Feraille, E., Carranza, M. L., Buffin-Meyer, B., Rousselot, M., Doucet, A., and Favre, H. (1995) Protein kinase C-dependent stimulation of Na(+)-K(+)-ATP epsilon in rat proximal convoluted tubules. *Am. J. Physiol.,* **268,** C1277–C1283.

Feraille, E., and Doucet, A. (2001) Hormonal control of Na,K-ATPase-dependant sodium transport in the kidney. *Physiol. Rev.,* **81,** 345–418.

Finotti, P., and Verbaro, R. (1987) Identification and partial purification of a Na,K-ATPase stimulating serine protease from plasma of insulin-dependent diabetics. *Clin. Chim. Acta,* **170,** 121–134.

Forbush, B., 3rd, Kaplan, J. H., and Hoffman, J. F. (1978) Characterization of a new photoaffinity derivative of ouabain: Labeling of the large polypeptide and of a proteolipid component of the Na,K-ATPase. *Biochemistry,* **17,** 3667–3676.

Forst, T., Dufayet de la Tour, D., Kunt, T., Pfutzner, A., Goitom, K., Pohlmann, T., Schneider, S., Johansson, B. L., Wahren, J., Lobig, M., Engelbach, M., Beyer, J., and Vague, P. (2000) The effect of C-peptide on cyclic GMP and erythrocyte Na/K ATPase activity in diabetes type I. *Clin. Sci.,* **98,** 283–290.

Gardner, K., and Bennett, V. (1986) A new erythrocyte membrane-associated protein with calmodulin binding activity. Identification and purification. *J. Biol. Chem.,* **261,** 1339–1348.

Geering, K. (2001) The functional role of beta subunits in oligomeric P-type ATPases. *J. Bioenerg. Biomembr.,* **33,** 425–438.

Gerbi, A., Barbey, O., Raccah, D., Coste, T., Jamme, I., Nouvelot, A., Ouafik, L., Levy, S., Vague, P., and Maixent, J. M. (1997) Alteration of Na,K-ATPase isoenzymes in diabetic cardiomyopathy: Effect of dietary supplementation with fish oil (n-3 fatty acids) in rats. *Diabetologia,* **40,** 496–505.

Gerbi, A., and Maixent, J. M. (1999) Fatty acid-induced modulation of ouabain responsiveness of rat Na,K-ATPase isoforms. *J. Membr. Biol.,* **168,** 19–27.

Gerbi, A., Maixent, J. M., Ansaldi, J. L., Pierlovisi, M., Coste, T., Pelissier, J. F., Vague, P., and Raccah, D. (1999) Fish oil supplementation prevents diabetes-induced nerve conduction velocity and neuroanatomical changes in rats. *J. Nutr.,* **129,** 207–213.

Giraud, F., Claret, M., Bruckdorfer, K. R., and Chailley, B. (1981) The effects of membrane lipid order and cholesterol on the internal and external cationic sites of the $Na^{+}$-$K^{+}$ pump in erythrocytes. *Biochim. Biophys. Acta,* **647,** 249–258.

Gnanaprakasam, M. S., and Srivastava, L. M. (1978) Effect of starvation, alloxan diabetes and adrenalectomy on $Na^{+}$ $K^{+}$- ATPase of the mucosa of the small intestine of rat. *Biochem. Exp. Biol.,* **14,** 257–262.

Green, R. J., King, R. H., Thomas, P. K., and Baron, D. N. (1985) Sodium-potassium-ATPase activity in the dorsal root ganglia of rats with streptozotocin-induced diabetes. *Diabetologia,* **28,** 104–107.

Greene, D. A., and Lattimer, S. A. (1983) Impaired rat sciatic nerve sodium-potassium adenosine triphosphatase in acute streptozocin diabetes and its correction by dietary *myo*-inositol supplementation. *J. Clin. Invest.,* **72,** 1058–1063.

Greene, D. A., Lattimer, S. A., and Sima, A. A. (1987) Sorbitol, phosphoinositides, and sodium-potassium-ATPase in the pathogenesis of diabetic complications. *N. Engl. J. Med.,* **316,** 599–606.

Greene, D. A., and Mackway, A. M. (1986) Decreased *myo*-inositol content and $Na^{+}$-$K^{+}$-ATPase activity in superior cervical ganglion of STZ-diabetic rat and prevention by aldose reductase inhibition. *Diabetes,* **35,** 1106–1108.

Hansen, A., Johansson, B. L., Wahren, J., and Von Bibra, H. (2002) C-peptide exerts beneficial effects on myocardial blood flow and function in patients with type 1 diabetes. *Diabetes,* **51,** 3077–3082.

Ido, Y., Vindigni, A., Chang, K., Stramm, L., Chance, R., Heat, W. F., DiMarchi, R. D., Di Cera, E., and Williamson, J. R. (1997) Prevention of vascular and neural dysfunction in diabetic rat by C-peptide. *Science,* **277,** 563–567.

Imanaga, I. (1976) Effects of insulin on mammalian cardiac muscle. *Recent Adv. Stud. Cardiac Struct. Metab.,* **11,** 441–450.

Issautier, T., Kovacic, H., Gallice, P., Raccah, D., Vague, P., and Crevat, A. (1994) Modulation defect of sodium pump evidenced in diabetic patients by a microcalorimetric study. *Clin. Chim. Acta,* **228,** 161–170.

Jannot, M. F., Raccah, D., De La Tour, D. D., Coste, T., and Vague, P. (2002) Genetic and environmental regulation of Na/K adenosine triphosphatase activity in diabetic patients. *Metabolism,* **51,** 284–291.

Johannson, A., Smith, G. A., and Metcalfe, J. C. (1981) The effect of bilayer thickness on the activity of Na,K-ATPase. *Biochem. Biophys. Acta,* **641,** 416–421.

Johansson, B.-L., Borg, K., Fernkvist-Forbes, E., Kernell, A., Odergren, T., and Wahren, J. (2000) Beneficial effects of C-peptide on incipient nephropathy and neuropathy in patients with type I diabetes-a three months study. *Diabet. Med.,* **17,** 181–189.

Johansson, B. L., Kernell, A., Sjoberg, S., and Wahren, J. (1993) Influence of combined C-peptide and insulin administration on renal function and metabolic control in diabetes type 1. *J. Clin. Endocrinol. Metab.,* **77,** 976–981.

Johansson, B. L., Linde, B., and Wahren, J. (1992) Effects of C-peptide on blood flow, capillary diffusion capacity and glucose utilization in the exercising forearm of type 1 (insulin-dependent) diabetic patients. *Diabetologia,* **35,** 1151–1158.

Johansson, J., Ekberg, K., Shafqat, J., Henriksson, M., Chibalin, A., Wahren, J., and Jornvall, H. (2002) Molecular effects of proinsulin C-peptide. *Biochem. Biophys. Res. Commun.,* **295,** 1035–1040.

Jorgensen, P. L. (1982) Mechanism of the $Na^+$,$K^+$ pump. Protein structure and conformations of the pure ($Na^+$+$K^+$)-ATPase. *Biochim. Biophys. Acta,* **694,** 27–68.

Juhan, I., Vague, P., Buonocore, M., Moulin, J. P., Jouve, R., and Vialettes, B. (1982) Abnormalities of erythrocyte deformability and platelet aggregation in insulin-dependent diabetics corrected by insulin in vivo and in vitro. *Lancet,* **1,** 535–537.

Kaplan, J. H. (1985) Ion movements through the sodium pump. *Annu. Rev. Physiol.,* **47,** 535–544.

Karim, Z., Defontaine, N., Paillard, M., and Poggioli, J. (1995) Protein kinase C isoforms in rat kidney proximal tubule: acute effect of angiotensin II. *Am. J. Physiol.,* **269,** C134–C140.

Kimelberg, H. K. (1975) Alterations in phospholipid-dependent ($Na^+$+$K^+$)-ATPase activity due to lipid fluidity. Effects of cholesterol and $Mg^{2+}$. *Biochim. Biophys. Acta,* **413,** 143–156.

Kimelberg, H. K., and Mayhew, E. (1975) Increased ouabain-sensitive 86$Rb^+$ uptake and sodium and potassium ion-activated adenosine triphosphatase activity in transformed cell lines. *J. Biol. Chem.,* **250,** 100–104.

Kimelberg, H. K., and Papahadjopoulos, D. (1972) Phospholipid requirements for ($Na^+ + K^+$)-ATPase activity: Head-group specificity and fatty acid fluidity. *Biochim. Biophys. Acta,* **282,** 277–292.

Kitamura, T., Kimura, K., Jung, B. D., Makondo, K., Okamoto, S., Canas, X., Sakane, N., Yoshida, T., and Saito, M. (2001) Proinsulin C-peptide rapidly stimulates mitogen-activated protein kinases in Swiss 3T3 fibroblasts: Requirement of protein kinase C, phosphoinositide 3-kinase and pertussis toxin-sensitive G-protein. *Biochem. J.,* **355,** 123–129.

Kjeldsen, K., Braendgaard, H., Sidenius, P., Larsen, J. S., and Norgaard, A. (1987) Diabetes decreases $Na^+$-$K^+$ pump concentration in skeletal muscles, heart ventricular muscle, and peripheral nerves of rat. *Diabetes,* **36,** 842–848.

Kotyk, A., and Amler, E. (1995) Na,K-adenosinetriphosphatase: The paradigm of a membrane transport protein. *Physiol. Res.,* **44,** 261–274.

Ku, D. D., Sellers, B. M., and Meezan, E. (1986) Development of renal hypertrophy and increased renal $Na^+$,$K^+$-ATPase in streptozotocin-diabetic rats. *Endocrinology,* **119,** 672–679.

Kunt, T., Schneider, S., Pfützner, A., Goitom, K., Engelbach, M., Schauf, B., Beyer, J., and Forst, T. (1999) The effect of human proinsulin C-peptide on erythocyte deformability in patients with type I diabetes mellitus. *Diabetologia,* **42,** 465–471.

La Rocca, E., Fiorina, P., Di Carlo, V., Astorri, E., Rossetti, C., Lucignani, G., Fazio, F., Giudici, D., Cristallo, M., Bianchi, G., Pozza, G., and Secchi, A. (2001) Cardiovascular outcomes after kidney-pancreas and kidney-alone transplantation. *Kidney Int.,* **60,** 1964–1971.

Lambourne, J. E., Brown, A. M., Calcutt, N., Tomlinson, D. R., and Willars, G. B. (1988) Adenosine triphosphatase in nerves and ganglia of rats with streptozotocin-induced diabetes or galactosaemia; effects of aldose reductase inhibition. *Diabetologia,* **31,** 379–384.

Lasker, N., Hopp, L., Grossman, S., Bamforth, R., and Aviv, A. (1985) Race and sex differences in erythrocyte $Na^+$, $K^+$, and $Na^+$-$K^+$-adenosine triphosphatase. *J. Clin. Invest.,* **75,** 1813–1820.

Leaf, A., and Weber, P. C. (1988) Cardiovascular effects of n-3 fatty acids. *N. Engl. J. Med.,* **318,** 549–557.

Leong, S. F., and Leung, T. K. (1991) Diabetes induced by streptozotocin causes reduced Na-K ATPase in the brain. *Neurochem. Res.,* **16,** 1161–1165.

Luppa, D., and Muller, F. (1986) Effect of diabetes and adrenocortical state on intestinal transport capacity and ($Na^+ + K^+$)-activated adenosine triphosphatase activity. *Diabete Metab.,* **12,** 191–196.

Mandon, B., Siga, E., Chabardes, D., Firsov, D., Roinel, N., and De Rouffignac, C. (1993) Insulin stimulates $Na^+$, $Cl^-$, $Ca^{2+}$, and $Mg^{2+}$ transports in TAL of mouse nephron: Cross-potentiation with AVP. *Am. J. Physiol.,* **265,** F361–F369.

Martiny-Baron, G., Kazanietz, M. G., Mischak, H., Blumberg, P. M., Kochs, G., Hug, H., Marme, D., and Schachtele, C. (1993) Selective inhibition of protein kinase C isozymes by the indolocarbazole Go 6976. *J. Biol. Chem.,* **268,** 9194–9197.

Mayanil, C. S., Kazmi, S. M., and Baquer, N. Z. (1982) $Na^+$,$K^+$-ATPase and $Mg^{2+}$-ATPase activities in different regions of rat brain during alloxan diabetes. *J. Neurochem.,* **39,** 903–908.

McDonough, A. A., Geering, K., and Farley, R. A. (1990) The sodium pump needs its beta subunit. *FASEB J.,* **4,** 1598–1605.

McGregor, E., Isles, C. G., Jay, J. L., Lever, A. F., and Murray, G. D. (1986) Retinal changes in malignant hypertension. *BMJ,* **292,** 233–234.

McKenna, K., Morris, A. D., Azam, H., Newton, R. W., Baylis, P. H., and Thompson, C. J. (1999) Exaggerated vasopressin secretion and attenuated osmoregulated thirst in human survivors of hyperosmolar coma. *Diabetologia,* **42,** 534–538.

McKenna, K., Morris, A. D., Ryan, M., Newton, R. W., Frier, B. M., Baylis, P. H., Saito, T., Ishikawa, S., and Thompson, C. J. (2000) Renal resistance to vasopressin in poorly controlled type 1 diabetes mellitus. *Am. J. Physiol. Endocrinol. Metab.,* **279,** E155–E160.

McLennan, P. L., Abeywardena, M. Y., and Charnock, J. S. (1985) Influence of dietary lipids on arrhythmias and infarction after coronary artery ligation in rats. *Can. J. Physiol. Pharmacol.,* **63,** 1411–1417.

McLennan, P. L., Abeywardena, M. Y., and Charnock, J. S. (1988) Dietary fish oil prevents ventricular fibrillation following coronary artery occlusion and reperfusion. *Am. Heart J.,* **116,** 709–717.

McMillan, D. E. (1975) Deterioration of the microcirculation in diabetes. *Diabetes,* **24,** 944–957.

Mercer, R. W. (1993) Structure of the Na,K-ATPase. *Int. Rev. Cytol.,* **137C,** 139–168.

Mizuno, K., Kato, N., Makino, M., Suzuki, T., and Shindo, M. (1999) Continuous inhibition of excessive polyol pathway flux in peripheral nerves by aldose reductase inhibitor fidarestat leads to improvement of diabetic neuropathy. *J. Diabetes Complications,* **13,** 141–150.

Mobasheri, A., Avila, J., Cozar-Castellano, I., Brownleader, M. D., Trevan, M., Francis, M. J., Lamb, J. F., and Martin-Vasallo, P. (2000) $Na^+$, $K^+$-ATPase isozyme diversity; comparative biochemistry and physiological implications of novel functional interactions. *Biosci. Rep.,* **20,** 51–91.

Modyanov, N. N., Petrukhin, K. E., Sverdlov, V. E., Grishin, A. V., Orlova, M. Y., Kostina, M. B., Makarevich, O. I., Broude, N. E., Monastyrskaya, G. S., and Sverdlov, E. D. (1991) The family of human Na,K-ATPase genes. ATP1AL1 gene is transcriptionally competent and probably encodes the related ion transport ATPase. *FEBS Lett.,* **278,** 91–94.

Ng, Y. C., Tolerico, P. H., and Book, C. B. (1993) Alterations in levels of Na(+)-K(+)-ATPase isoforms in heart, skeletal muscle, and kidney of diabetic rats. *Am. J. Physiol.,* **265,** E243–E251.

Nishida, K., Ohara, T., Johnson, J., Wallner, J. S., Wilk, J., Sherman, N., Kawakami, K., Sussman, K. E., and Draznin, B. (1992) $Na^+$/K(+)-ATPase activity and its alpha II subunit gene expression in rat skeletal muscle: Influence of diabetes, fasting, and refeeding. *Metabolism,* **41,** 56–63.

Nowak, T. V., Castelaz, C., Ramaswamy, K., and Weisbruch, J. P. (1995) Impaired rodent vagal nerve sodium-potassium-ATPase activity in streptozotocin diabetes. *J. Lab. Clin. Med.,* **125,** 182–186.

Ohara, T., Sussman, K. E., and Draznin, B. (1991) Effect of diabetes on cytosolic free $Ca^{2+}$ and Na(+)-K(+)-ATPase in rat aorta. *Diabetes,* **40,** 1560–1563.

Ohtomo, Y., Aperia, A., Sahlgren, B., Johansson, B. L., and Wahren, J. (1996) C-peptide stimulates rat renal tubular $Na^+$, K(+)-ATPase activity in synergism with neuropeptide Y. *Diabetologia,* **39,** 199–205.

Ohtomo, Y., Bergman, T., Johansson, B. L., Jornvall, H., and Wahren, J. (1998) Differential effects of proinsulin C-peptide fragments on $Na^+$, $K^+$-ATPase activity of renal tubule segments. *Diabetologia,* **41,** 287–291.

Pierce, G. N., and Dhalla, N. S. (1983) Sarcolemmal $Na^+$-$K^+$-ATPase activity in diabetic rat heart. *Am. J. Physiol.,* **245,** C241–C247.

Raccah, D., Dadoun, F., Coste, T., and Vague, P. (1996a) Decreased Na/K ATPase ouabain binding sites in red blood cells of patients with insulin-dependent diabetes and healthy north African control subjects: Relationship with diabetic neuropathy. *Horm. Metab. Res.,* **28,** 128–132.

Raccah, D., Fabreguettes, C., Azulay, J. P., and Vague, P. (1996b) Erythrocyte Na(+)-K(+)-ATPase activity, metabolic control, and neuropathy in IDDM patients [published erratum appears in *Diabetes Care* 1997 Feb; 20(2):236]. *Diabetes Care,* **19,** 564–568.

Raccah, D., Gallice, P., Pouget, J., and Vague, P. (1992) Hypothesis: Low Na/K-ATPase activity in the red cell membrane, a potential marker of the predisposition to diabetic neuropathy. *Diabete Metab.,* **18,** 236–241.

Rahmani-Jourdheuil, D., Mourayre, Y., Vague, P., Boyer, J., and Juhan-Vague, I. (1987) In vivo insulin effect on ATPase activities in erythrocyte membrane from insulin-dependent diabetics. *Diabetes,* **36,** 991–995.

Rigler, R., Pramanik, A., Jonasson, P., Kratz, G., Jansson, O. T., Nygren, P.-A., Stahl, S., Ekberg, K., Johansson, B. L., Uhlen, S., Uhlen, M., Jornvall, H., and Wahren, J. (1999) Specific binding of the proinsulin C-peptide to human cell membranes. *Proc. Natl. Acad. Sci. U. S. A.,* **96,** 13318–13323.

Samnegard, B., Jacobson, S. H., Jaremko, G., Johansson, B. L., and Sjoquist, M. (2001) Effects of C-peptide on glomerular and renal size and renal function in diabetic rats. *Kidney Int.,* **60,** 1258–1265.

Scarpini, E., Bianchi, R., Moggio, M., Sciacco, M., Fiori, M. G., and Scarlato, G. (1993) Decrease of nerve Na/K ATPase activity in the pathogenesis of human diabetic neuropathy. *J. Neurol. Sci.,* **120,** 159–167.

Schaffer, S. W. (1991) Cardiomyopathy associated with noninsulin-dependent diabetes. *Mol. Cell. Biochem.,* **107,** 1–20.

Scherzer, P., and Popovtzer, M. M. (2002) Segmental localization of mRNAs encoding Na(+)-K(+)-ATPase alpha(1)- and beta(1)-subunits in diabetic rat kidneys using RT-PCR. *Am. J. Physiol. Renal Physiol.,* **282,** F492–F500.

Schmid-Schonbein, H., and Volger, E. (1976) Red-cell aggregation and red-cell deformability in diabetes. *Diabetes,* **25,** 897–902.

Sennoune, S., Gerbi, A., Duran, M. J., Grillasca, J. P., Compe, E., Pierre, S., Planells, R., Bourdeau, M., Vague, P., Pieroni, G., and Maixent, J. M. (2000) Effect of streptozotocin-induced diabetes on rat liver $Na^+$/$K^+$-ATPase. *Eur. J. Biochem.,* **267,** 1–9.

Shafqat, J., Juntti-Berggren, L., Zhong, Z., Ekberg, K., Kohler, M., Berggren, P. O., Johansson, J., Wahren, J., and Jornvall, H. (2002) Proinsulin C-peptide and its analogues induce intracellular $Ca^{2+}$ increases in human renal tubular cells. *Cell. Mol. Life Sci.,* **59,** 1185–1189.

Shamraj, O. I., and Lingrel, J. B. (1994) A putative fourth $Na^+$,K(+)-ATPase alpha-subunit gene is expressed in testis. *Proc. Natl. Acad. Sci. U. S. A.,* **91,** 12952–12956.

Shull, M. M., Pugh, D. G., and Lingrel, J. B. (1990) The human Na, K-ATPase alpha 1 gene: Characterization of the 5′- flanking region and identification of a restriction fragment length polymorphism. *Genomics,* **6,** 451–460.

Sima, A. A., and Sugimoto, K. (1999) Experimental diabetic neuropathy: An update. *Diabetologia,* **42,** 773–788.

Sima, A. A., Zhang, W., Sugimoto, K., Henry, D., Li, Z., Wahren, J., and Grunberger, G. (2001) C-peptide prevents and improves chronic type I diabetic polyneuropathy in the BB/Wor rat. *Diabetologia,* **44,** 889–897.

Sima, A. A., Zhang, W., Xu, G., Sugimoto, K., Guberski, D., and Yorek, M. A. (2000) A comparison of diabetic polyneuropathy in type II diabetic BBZDR/Wor rats and in type I diabetic BB/Wor rats. *Diabetologia,* **43,** 786–793.

Sjoquist, M., Huang, W., and Johansson, B. L. (1998) Effects of C-peptide on renal function at the early stage of experimental diabetes. *Kidney Int.,* **54,** 758–764.

Sonnenblick, E. H., Fein, F., Capasso, J. M., and Factor, S. M. (1985) Microvascular spasm as a cause of cardiomyopathies and the calcium- blocking agent verapamil as potential primary therapy. *Am. J. Cardiol.,* **55,** 179B–184B.

Sweadner, K. J. (1989) Isozymes of the $Na^+$/$K^+$-ATPase. *Biochim. Biophys. Acta,* **988,** 185–220.

Sweeney, G., and Klip, A. (1998) Regulation of the $Na^+$/$K^+$-ATPase by insulin: Why and how? *Mol. Cell. Biochem.,* **182,** 121–133.

Therien, A. G., Goldshleger, R., Karlish, S. J., and Blostein, R. (1997) Tissue-specific distribution and modulatory role of the gamma subunit of the Na,K-ATPase. *J. Biol. Chem.,* **272,** 32628–32634.

Trinh-Trang-Tan, M. M., Bankir, L., Doucet, A., el Mernissi, G., Imbert-Teboul, M., Montegut, M., Siaume, S., and Morel, F. (1985) Influence of chronic ADH treatment on adenylate cyclase and ATPase activity in distal nephron segments of diabetes insipidus Brattleboro rats. *Pflugers Arch.,* **405,** 216–222.

Tsimaratos, M., Coste, T. C., Djemli-Shipkolye, A., Daniel, L., Shipkolye, F., Vague, P., and Raccah, D. (2001) Evidence of time-dependent changes in renal medullary Na,K-ATPase activity and expression in diabetic rats. *Cell. Mol. Biol.,* **47,** 239–245.

Tsimaratos, M., Roger, F., Mordasini, D., Hasler, U., Martin, P.-Y., and Feraille, E. (2003) C-peptide stimulates Na,K-ATPase activity via PKC alpha in rat medullary thick ascending limb. *Diabetologia,* **46,** 124–131.

Tucker, B. J., Rasch, R., and Blantz, R. C. (1993) Glomerular filtration and tubular reabsorption of albumin in preproteinuric and proteinuric diabetic rats. *J. Clin. Invest.,* **92,** 686–694.

Vague, P., Brunetti, O., Valet, A. M., Attali, I., Lassmann-Vague, V., and Vialettes, B. (1988) Increased prevalence of neurologic complications among insulin dependent diabetic patients of Algerian origin. *Diabetes Metab.,* **14,** 706–711.

Vague, P., Dufayet, D., Coste, T., Moriscot, C., Jannot, M. F., and Raccah, D. (1997a) Association of diabetic neuropathy with Na/K ATPase gene polymorphism. *Diabetologia,* **40,** 506–511.

Vague, P., Dufayet, D., Lamotte, M. F., Mouchot, C., and Raccah, D. (1997b) [Genetic factors, Na K ATPase activity and neuropathy in diabetics]. *Bull. Acad. Natl. Med.,* **181,** 1811–1821; discussion 1821–1823.

Vague, P., and Juhan, I. (1983) Red cell deformability, platelet aggregation, and insulin action. *Diabetes,* **32(Suppl 2),** 88–91.

Ver, A., Csermely, P., Banyasz, T., Kovacs, T., and Somogyi, J. (1995a) Alterations in the properties and isoform ratios of brain $Na^+/K(+)$-ATPase in streptozotocin diabetic rats. *Biochim. Biophys. Acta,* **1237,** 143–150.

Ver, A., Szanto, I., Banyasz, T., Csermely, P., Vegh, E., and Somogyi, J. (1997) Changes in the expression of $Na^+/K^+$-ATPase isoenzymes in the left ventricle of diabetic rat hearts: Effect of insulin treatment. *Diabetologia,* **40,** 1255–1262.

Ver, A., Szanto, I., Csermely, P., Kalff, K., Vegh, E., Banyasz, T., Marcsek, Z., Kovacs, T., and Somogyi, J. (1995b) Effect of streptozotocin-induced diabetes on kidney $Na^+/K(+)$-ATPase. *Acta Physiol. Hung.,* **83,** 323–332.

Wald, H., and Popovtzer, M. M. (1984) The effect of streptozotocin-induced diabetes mellitus on urinary excretion of sodium and renal $Na^+$-$K^+$-ATPase activity. *Pflugers Arch.,* **401,** 97–100.

Weaver, B. J., and Holub, B. J. (1987) The thrombin-dependent enrichment of alkenylacyl ethanolamine phosphoglyceride with [14C]eicosapentaenoic and [3H]arachidonic acids in prelabelled human platelets. *Biochem. Cell. Biol.,* **65,** 405–408.

Wright, R. A., and Nukada, H. (1994) Vascular and metabolic factors in the pathogenesis of experimental diabetic neuropathy in mature rats. *Brain,* **117,** 1395–1407.

Yang-Feng, T. L., Schneider, J. W., Lindgren, V., Shull, M. M., Benz, E. J., Jr., Lingrel, J. B., and Francke, U. (1988) Chromosomal localization of human $Na^+$, $K^+$-ATPase alpha- and beta-subunit genes. *Genomics,* **2,** 128–138.

Yorek, M. A., Dunlap, J. A., and Ginsberg, B. H. (1988) Effect of increased glucose levels on $Na^+/K^+$-pump activity in cultured neuroblastoma cells. *J. Neurochem.,* **51,** 605–610.

Zerbe, R. L., Vinicor, F., and Robertson, G. L. (1979) Plasma vasopressin in uncontrolled diabetes mellitus. *Diabetes,* **28,** 503–508.

